# Evaluation of 2D Imaging Schemes for Pulsed Arterial Spin Labeling of the Human Kidney Cortex

**DOI:** 10.3390/diagnostics8030043

**Published:** 2018-06-28

**Authors:** Charlotte E. Buchanan, Eleanor F. Cox, Susan T. Francis

**Affiliations:** Sir Peter Mansfield Imaging Centre, School of Physics and Astronomy, University of Nottingham, Nottingham NG7 2RD, UK; charlotte.buchanan@nottingham.ac.uk (C.E.B.); eleanor.cox@nottingham.ac.uk (E.F.C.)

**Keywords:** magnetic resonance imaging (MRI), arterial spin labeling, renal MRI, perfusion, renal ASL

## Abstract

A number of imaging readout schemes are proposed for renal arterial spin labeling (ASL) to quantify kidney cortex perfusion, including gradient echo-based methods of balanced fast field echo (bFFE) and gradient-echo echo-planar imaging (GE-EPI), or spin echo-based schemes of spin-echo echo-planar imaging (SE-EPI) and turbo spin-echo (TSE). Here, we compare these two-dimensional (2D) imaging schemes to evaluate the optimal imaging scheme for pulsed ASL (PASL) assessment of human kidney cortex perfusion at 3 T. Ten healthy volunteers with normal renal function were scanned using each 2D multi-slice imaging scheme, in combination with a respiratory triggered flow-sensitive alternating inversion recovery (FAIR) ASL scheme on a 3 T Philips Achieva scanner. All volunteers returned for a second identical scan session within two weeks of the first scan session. Comparisons were made between the imaging schemes in terms of perfusion-weighted image (PWI) signal-to-noise ratio (SNR) and perfusion quantification, temporal SNR (tSNR), spatial coverage, and repeatability. For each imaging scheme, the renal cortex perfusion was calculated (bFFE: 276 ± 29 mL/100g/min, GE-EPI: 222 ± 18 mL/100g/min, SE-EPI: 201 ± 36 mL/100g/min, and TSE: 200 ± 20 mL/100g/min). Perfusion was found to be higher for GE-based readouts when compared with SE-based readouts, with significantly higher measured perfusion for the bFFE readout when compared with all other schemes (*p* < 0.05), attributed to the greater vascular signal present. Despite the PWI-SNR being significantly lower for SE-EPI when compared with all other schemes (*p* < 0.05), the SE-EPI readout gave the highest tSNR, and was found to be the most reproducible scheme for the assessment of kidney cortex, with a coefficient of variation (CoV) of 17.2%, whilst minimizing variability of the perfusion-weighted signal across slices for whole-kidney perfusion assessment. For the assessment of kidney cortex perfusion using 2D readout schemes, SE-EPI provides optimal tSNR, minimal variability across slices, and repeatable data acquired in a short scan time with low specific absorption rate.

## 1. Introduction

In clinical practice, renal function is typically determined via serum creatinine measurements to estimate glomerular filtration rate (GFR); however, this method is not highly sensitive, and changes in GFR may develop relatively late in the progression of chronic kidney disease (CKD). Renal perfusion informs on the delivery of nutrients and oxygen to the tissue, and is a key measure by which to monitor renal function. A method of providing reliable and repeatable perfusion assessment of the kidney, in conjunction with precise morphological information, would significantly improve the assessment and monitoring of renal health. Arterial spin labeling (ASL) is a magnetic resonance imaging (MRI) technique that allows the non-invasive quantitative assessment of tissue perfusion, with the advantage of not requiring any exogenous contrast agent, instead using the magnetization of endogenous-labeled blood to provide contrast.

The majority of renal ASL studies in the literature employed a pulsed ASL (PASL) technique using the flow-sensitive alternating inversion recovery (FAIR) scheme [1,2,3,4,5,6,7,8]. In the FAIR scheme, two images are collected, a selective image which contains non-inverted arterial blood and a non-selective image in which inflowing blood is magnetically inverted. By subtracting the non-selective image from the selective image, a perfusion-weighted image (PWI) is formed, which with the appropriate modeling can be quantified using a perfusion map in units of mL/100g/min.

A number of different two-dimensional (2D) imaging readout schemes were implemented in the literature for renal ASL studies. To determine the optimal readout for renal ASL, a number of factors must be considered. The optimal readout should have a short echo time (TE) in order to provide the highest image signal-to-noise ratio (SNR), and to reduce the amount of signal dephasing and distortion. The short intrinsic T_2_ and T_2_* in the abdomen lead to rapid signal dropout and the loss of perfusion signal at longer TEs. The ideal readout should be collected in a short shot length to enable multiple slices through the kidney to be acquired prior to the decay of the ASL label, thus enabling whole-kidney perfusion assessment. Furthermore, if the acquisition is respiratorily triggered, it is important to acquire all images within a respiratory cycle, and ideally within the flat component of the respiratory cycle at end expiration where motion is minimal. Finally, the ideal readout should have a low specific absorption rate (SAR) so that a short temporal spacing between the 2D images can be achieved when collecting a multi-slice dataset.

Echo planar imaging (EPI) is one of the most commonly used readout techniques for ASL of the brain due to its relatively short acquisition time making whole-head coverage feasible, and its applicability is used and discussed in References [9,10,11,12,13,14,15,16]. However, in the body, the larger field of view (FOV) means EPI readouts typically have a longer TE (>10 ms, dependent on the parallel imaging acceleration factor), and for high-spatial-resolution images, the acquisition time can become very long resulting in poor image quality due to susceptibility-induced signal inhomogeneities, particularly close to geometrically irregular tissue–air boundaries. Either gradient echo (GE)- or spin-echo (SE)-based EPI can be used. GE-EPI is more influenced by any B_0_ field homogeneity than SE-EPI, since phase shifts from field inhomogeneities, static tissue susceptibility gradients, and chemical shifts are not cancelled. Since EPI has a short acquisition time, of the order of 30 ms, multiple slices at the peak of the ASL signal curve can be imaged; thus, low variance is expected in the perfusion-weighted ASL signal across a multi-slice dataset. Sokolska et al. used a GE-EPI readout combined with pseudo-continuous ASL (pCASL) labeling to assess the feasibility and within-subject repeatability of renal perfusion measures [17], whilst Gardener et al. employed a SE-EPI readout to acquire multiple slices across the kidney, and assessed different breathing strategies to overcome respiratory motion [4].

A balanced fast field echo scheme (bFFE), is widely used as the image readout for renal ASL [3,5,6,18]. It has the advantage of providing a very short TE and high image SNR. However, the shot length of a bFFE scheme is long, at approximately 300 ms for a typical abdominal FOV with 3 mm voxel resolution. Thus, for a multi-slice acquisition, not all slices are collected at the peak of the ASL signal curve, potentially resulting in greater variance in the image perfusion-weighted signal across slices in comparison to EPI. In addition, the long shot length limits the number of slices which can be collected when respiratorily triggering the data acquisition. The bFFE schemes are also limited by their sensitivity to field inhomogeneity, with banding artefacts apparent in areas of off-resonance in the image. Gillis et al. measured inter-study reproducibility of ASL at 3 T using a FAIR scheme combined with bFFE readout, and concluded that this provides a repeatable method of measuring renal perfusion [5].

Turbo spin-echo (TSE) imaging, also known as fast spin-echo (FSE) imaging, is another alternative spin-echo-based 2D imaging scheme. Here, the time saved by scanning multiple lines of k-space at once, when compared with standard spin-echo imaging, means it is possible to lengthen the time between each excitation pulse, thus allowing more time for T_1_ recovery within the readout scheme, and thus, resulting in improved image SNR. A higher number of phase-encoding steps can also be used for improved spatial resolution, and susceptibility-induced signal losses are low. However, the TE of a TSE readout is typically 50 ms, so some blurring of the image will occur due to T_2_ decay, whilst the shot length is long at approximately 160 ms, resulting in slices being acquired at different points in the ASL signal curve and respiratory cycle, and therefore, potentially increasing the variance in the perfusion-weighted signal across slices. A further limitation of the TSE scheme is the high SAR due to the multiple refocusing pulses which can result in a long temporal spacing between multi-slice images to keep within SAR limits.

It should be noted that three-dimensional (3D) readout schemes are applied to renal ASL. In References [1,19], a 3D GRASE scheme was applied to assess whole-kidney perfusion in healthy volunteers. In a more recent publication, a TSE acquisition was used to evaluate 3D volumetric, isotropic-resolution renal ASL [20].

To date, there are no direct comparisons of these 2D imaging readout schemes for ASL in the kidney. In this work, we compared GE-EPI, SE-EPI, bFFE (a balanced gradient echo scheme also termed TrueFISP or FIESTA), and single-shot TSE (SSTSE) (a fast spin-echo sequence also termed fast SE (FSE)) readout schemes used in combination with a FAIR labeling scheme to assess the optimal scheme(s) for renal ASL.

## 2. Materials and Methods

### 2.1. Subjects

The study was approved by the local ethics committee, and all participants gave informed, written consent. Ten healthy volunteers (age 27 ± 10 years; five female) were scanned for approximately one hour on a 3 T Philips Achieva MRI scanner using dual-transmit and a 16-channel XLTorso receive coil. To assess the repeatability of each readout scheme, volunteers returned for a second visit, which comprised an identical scan session within two weeks of the first visit scan session. The MRI was performed at the same time of day on all subjects to minimize potential diurnal variations in renal physiologic function. Subjects fasted the previous evening from 8 pm to enable a controlled hydration status for all subjects. To ensure that all volunteers had normal kidney function, blood and urine samples were collected and evaluated by a clinician. Urea, electrolytes, and urine protein creatinine ratio were assessed.

### 2.2. MRI Acquisition

**Figure 1:**
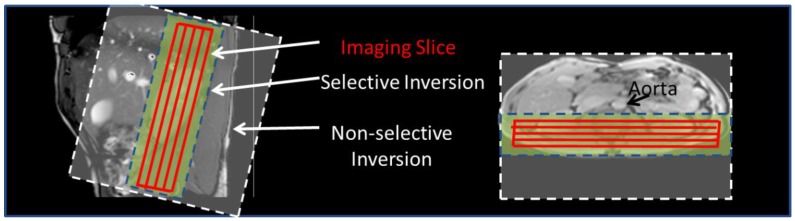
Flow-sensitive alternating inversion recovery (FAIR) scheme. Positioning of the selective and non-selective labeling slabs shown relative to the imaging volume of the kidneys and the aorta.

Initially, bFFE localizer scans were acquired in three orthogonal planes to plan placement of the imaging and ASL-labeling slabs relative to the kidneys and vessels. The FAIR labeling scheme used a frequency-offset corrected inversion (FOCI) pulse to achieve a 45 mm selective (S) inversion slab (10 mm wider than the imaging volume) and a 400 mm non-selective (NS) inversion slab. Using such a labeling slab means that the trailing edge of the non-selective label does not arrive within the label delay time. Coronal-oblique imaging slices were collected through the kidneys in descending order (lateral–medial) whilst taking care that the selective inversion slab avoided the aorta (Figure 1). Identical readout geometry was acquired on each subject for all imaging schemes, with a 288 × 288 mm FOV, in-plane spatial resolution of 3 mm and 5 mm slice thickness. All readout schemes were acquired with parallel acceleration with a SENSE factor of 2, thus reducing the achievable GE-EPI and SE-EPI TE, and minimizing the readout duration, thereby limiting susceptibility-related distortions and signal drop out, and allowing multiple slices to be acquired to sample the peak of the ASL signal curve.

To suppress any static tissue signal in the perfusion-weighted images, in-plane water suppression enhanced through T_1_ effects (WET) presaturation pulses were applied immediately prior to each S/NS pulse and a sinc post-saturation pulse was applied immediately after. A post-label delay (PLD equivalent to inversion time, TI), defined to be the time between the inversion pulse to the central k-space of the first slice, of 1300 ms was used for bFFE and TSE readouts, and 1800 ms for GE-EPI and SE-EPI readouts. This accounted for the different readout duration of each of the schemes (see Table 1), ensuring the maximum perfusion-weighted signal was sampled in the central slice for each scheme. All datasets were acquired respiratorily triggered on the S/NS RF pulse, with a minimum repetition time (TR) of 3 s between each S/NS RF pulse. In total, 25 S/NS image pairs were acquired for each readout scheme.

Base magnetization (M_0_) and T_1_ relaxation time images were each acquired with geometry and readout schemes matched to the ASL scheme to allow perfusion quantification. Base magnetization (M_0_) images were acquired at the same point in the respiratory cycle as the ASL data using a trigger delay matched to the ASL PLD time. A modified respiratorily triggered inversion-recovery sequence was implemented to map the T_1_ relaxation time in the renal cortex. Images were acquired at multiple inversion times (TI) of 200 ms to 1500 ms in 100 ms steps, but with all TIs collected in the respiratory cycle at the same time as the ASL data by introducing an additional delay (Tv) following the respiratory trigger, and prior to the inversion pulse [21]. T_1_ data were collected with a minimum TR of 8 s to allow full signal recovery using a 400 mm NS inversion slab. For GE-EPI and SE-EPI readouts, the multi-slice T_1_ dataset was acquired in descending order, while for the bFFE and TSE readout schemes, the multi-slice T_1_ dataset was acquired for both ascending and descending orders to increase the dynamic range of TI values [22].

### 2.3. Quantification of Renal Cortex Perfusion and T_1_

Analysis was performed using custom-written MATLAB programs (Matlab version 8.1, The MathWorks, Inc., Natick, MA, USA). ASL perfusion-weighted (PW) difference images were formed by subtracting the non-selective images from the selective images [21]. PW difference images were inspected for motion, misaligned pairs discarded, and the remaining PW difference images averaged to form an average PW difference image (ΔM) for each slice. These were then normalized to the base M_0_ image. T_1_ maps were formed by fitting the inversion recovery data to a two-parameter model. ΔM, T_1_, and base M_0_ maps were used to generate a renal perfusion (*f*) map, in units of mL/100g/min, by fitting the data to a kinetic model [23]. Each slice was fitted taking into account the exact post-label delay at which the slice was collected following the labeling pulse (see Table 1; i.e., 1300 ms, 1580 ms, 1860 ms, 2140 ms, and 2420 ms for slices 1 to 5 of the bFFE scheme), and assuming an arterial transit delay of 400 ms (as defined from previous healthy volunteer data [21]). To segment the renal cortex, the kidneys were manually segmented before a histogram of kidney T_1_ values was produced along with a threshold to create a cortex mask. This analysis procedure is outlined in Figure 3 of Reference [21] from which the mode of the distribution of perfusion values within the renal cortex could be computed. The renal cortex masks were compared across the readout schemes to ensure approximately the same number of voxels were assessed; the average DICE similarity coefficient between visits was calculated as 0.53 ± 0.13. The mean and standard deviation of perfusion values in the renal cortex were calculated for both left and right kidneys across subjects.

### 2.4. Image Quality Assessment

The following quantitative metrics were computed in the renal cortex to assess the quality of perfusion data for each readout scheme: (i) perfusion-weighted image (PWI) SNR, defined as the mean PW signal divided by the standard deviation in the background noise of the PW image; (ii) temporal SNR (tSNR) of the perfusion-weighted image, defined as the mean PW signal divided by the standard deviation across the 25 ASL pairs (average across slices); and (iii) variance in the PW signal across slices (var_ΔM_), defined as the standard deviation in the PW signal across the slices divided by the mean PW signal.

### 2.5. Statistical Analysis

Statistical analysis was performed using SPSS software version 21(IBM©). Quantitative variables are expressed as mean ± standard deviation (SD) or median and interquartile range (IQR) depending on normality, with a Shapiro–Wilk test used to test for normality of the data. In all analyses, a *p*-value < 0.05 was considered as statistically significant. To assess differences between readout schemes, a repeated measures ANOVA test was used.

For each readout, the between- and within-subject variability of measurements was assessed by the coefficient of variation (bCV, wCV). In addition, the coefficient of variation (CoV) of perfusion (standard deviation divided by the mean) was calculated to assess repeatability between scan sessions.

## 3. Results

All healthy volunteers were confirmed to have normal kidney function, with eGFR > 60 mL/min/1.73 m^2^, with a creatinine level of 76 ± 15 μmol/L, and a urea level of 4.2 ± 1.1 mmol/L.

### 3.1. ASL Image Quality

**Figure 2:**
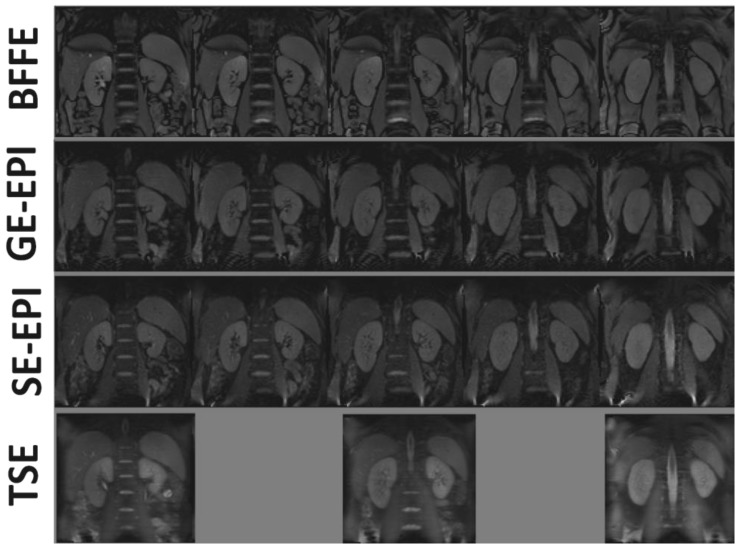
Example base magnetization (M_0_) images for the balanced fast field echo (bFFE), gradient-echo echo-planar imaging (GE-EPI), spin-echo echo-planar imaging (SE-EPI), and turbo spin-echo (TSE) readout schemes.

Base M_0_ images for each 2D readout scheme are shown in Figure 2, with good data quality for all readouts and minimal distortions, even for EPI acquisitions. Note that the TSE scheme suffered from blurring, whilst vessels appeared brighter in the bFFE image.

The TSE ASL scheme had the highest SAR at approximately 70% of the whole-body averaged SAR, whilst the SE-EPI, GE-EPI, and bFFE ASL schemes all had SAR values of less than 35%. To minimize SAR, the TSE scheme implements a longer temporal spacing between slice acquisitions (see Table 1), limiting the TSE acquisition to three slices acquired at the peak of the ASL signal curve.

**Figure 3:**
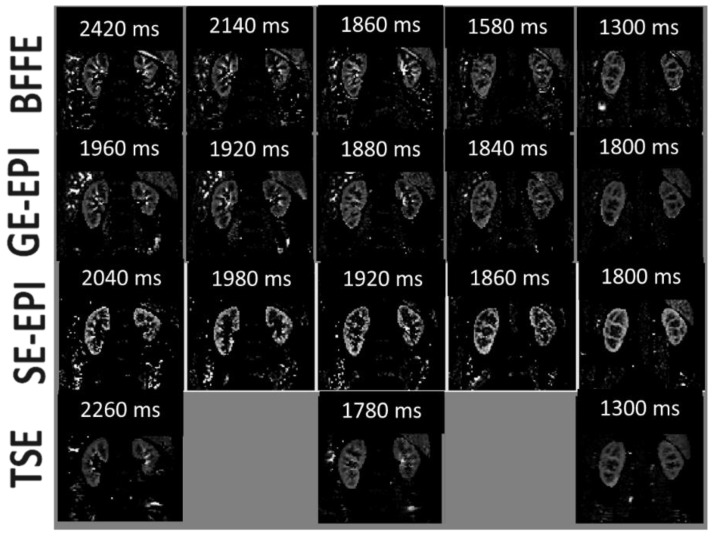
Example perfusion-weighted images (PWI) for each scheme (bFFE, GE-EPI, SE-EPI, and TSE) from a single subject. The post-label delay of each slice is indicated on each image.

**Figure 4:**
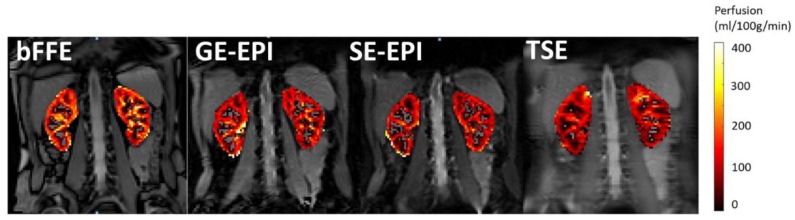
Example perfusion maps for each readout scheme (bFFE, GE-EPI, SE-EPI, and single-shot TSE) from a single subject shown for the central slice. Note the higher perfusion signal of the bFFE scheme due to contributions from the arcuate arteries of the kidney.

Figure 3 shows multi-slice average perfusion-weighted images for each readout scheme. The tSNR, PWI-SNR, and variability of the perfusion-weighted signal (var_∆M_) are provided in Table 2. The TSE scheme had the highest PWI-SNR, whilst the SE-EPI had the lowest PWI-SNR. However, tSNR was optimal for the SE-EPI scheme, whilst the GE-EPI scheme had the lowest t-SNR. The variability of the perfusion-weighted signal across slices (var_ΔM_) was found to be smallest for the SE-EPI scheme, reflecting this to be a good scheme for multi-slice whole kidney assessment, with the highest variability recorded for the bFFE scheme. Figure 4 shows example perfusion maps for each readout scheme.

### 3.2. Perfusion Quantification

**Figure 5:**
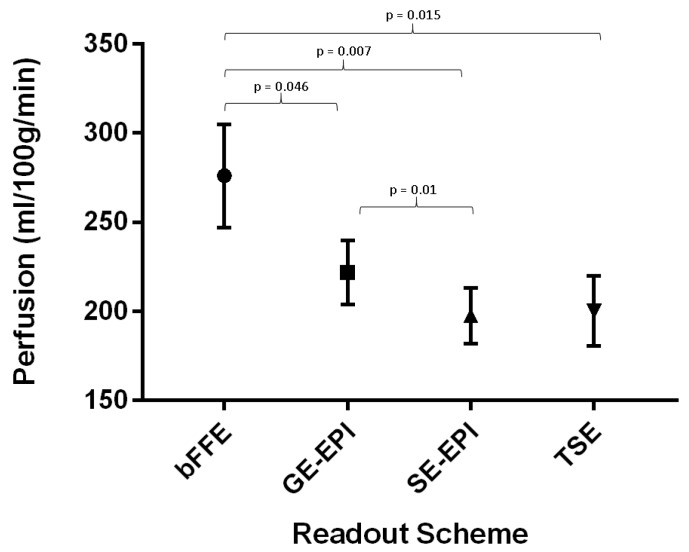
Renal cortical perfusion values measured from each readout scheme for the first visit. Values shown are the mean perfusion values with error bars showing the standard error of the mean. The bFFE readout gave significantly higher perfusion values than the other readouts (repeated measures ANOVA, *p* = 0.03). A significant difference was observed between SE-EPI and GE-EPI., with *p*-values of post-hoc paired *t*-tests shown.

Mean renal cortex perfusion across all readout schemes was 223 ± 11 mL/100g/min; the error indicates the standard error of the perfusion values across readout schemes. For each readout scheme, the measured renal cortex perfusion values for the first visit are shown in Figure 5, and were found to be as follows: bFFE—276 ± 29 mL/100g/min, GE-EPI—222 ± 18 mL/100g/min, SE-EPI—201 ± 36 mL/100g/min, and TSE—200 ± 20 mL/100g/min. A repeated measures ANOVA showed that the bFFE readout produced consistently higher perfusion values when compared with the other three schemes (*p* = 0.03), but also had the largest variance between subjects. The SE-EPI scheme gave the lowest within-subject variance.

The between-subject variation (bCV) of the schemes was 53.3%, 23.7%, 26.2%, and 35.9% for bFFE, GE-EPI, SE-EPI, and TSE, respectively. The within-subject variation (wCV) was 18.8% for bFFE, 23.9% for GE-EPI, 15.1% for SE-EPI, and 17.2% for TSE.

For each readout scheme, T_1_ of the renal cortex was also assessed and computed to be 1186 ± 147 ms, 1259 ± 214 ms, 1294 ± 168 ms, and 1135 ± 149 ms for bFFE, GE-EPI, SE-EPI, and TSE readout schemes, respectively.

### 3.3. Repeatability

The most repeatable readout scheme was SE-EPI, which had a CoV of 17.2%; the least repeatable scheme was the GE-EPI scheme with a CoV of 28.3%. For each readout scheme, across the 10 subjects, there were no significant differences in renal cortex perfusion values between the first and second visit (*p* > 0.05).

## 4. Discussion

Previous renal ASL studies used a variety of readout schemes in combination with FAIR labeling. In this work, a comparison of balanced fast field echo (bFFE), gradient-echo EPI (GE-EPI), spin-echo EPI (SE-EPI), and turbo spin-echo (TSE) schemes was made for renal ASL. For all schemes, multi-slice coverage could be achieved with five contiguous slices collected for bFFE, GE-EPI, and SE-EPI, and three slices with a 5 mm slice gap for TSE. The higher SAR led to wider readout spacing for the TSE scheme. GE-EPI and SE-EPI could achieve whole-kidney coverage in the shortest amount of time.

In this work, the cortical perfusion was higher for gradient-echo schemes (GE-EPI and bFFE) in comparison to spin-echo based schemes (SE-EPI and TSE). In particular, perfusion calculated from bFFE readout data was found to be significantly higher than that from all other schemes which could be attributed to the presence of vascular signal in these images. The bFFE scheme had the highest intravascular signal contribution of all readout schemes due to the high signal intensity from blood, which has an intrinsically high T_2_/T_1_ ratio, as well as the continuous replenishment of fresh blood and the fact that the longitudinal magnetization of flowing blood is minimally disturbed by the alpha pulse train in the bFFE readout scheme [24].

When comparing the SNR, the SE-EPI scheme gave a significantly lower PWI-SNR when compared with the other schemes; however, this could, in part, be attributed to the lower echo time of this sequence or lower contribution from vascular signal in the SE-EPI images. The SE-EPI scheme also gave the highest temporal SNR, suggesting lowest fluctuations from pulsatile vessels.

The SE-EPI scheme gave the lowest variance in perfusion-weighted signal (var_ΔM_) across slices of all the readout schemes. This is unsurprising as the SE-EPI scheme has a short shot length per slice, and so, all slices are acquired at almost the same point on both the ASL signal curve and in the respiratory cycle, resulting in a small variance in the signal of each slice. Conversely, TSE readouts have a long shot length; this yielded the highest variance in signal across slices.

All 2D readout schemes were determined to be repeatable with a CoV of 28.2% or less. SE-EPI was found to be optimal with a CoV of 17.2%. The ASL quantitative measurements of normal perfusion showed good within-subject variability and repeatability. Note that differences in distortions between the readout schemes are likely partly reflected in the DICE coefficients between the masks.

In addition, in this study, T_1_ maps were generated for each readout scheme, and used in the perfusion quantification, with mean T_1_ values of the renal cortex also computed. As expected, the bFFE and TSE readout schemes led to a shorter measured longitudinal recovery time when compared with EPI methods, likely due to the influence of T_2_*/T_2_ for the bFFE readout scheme [25], and the contribution of magnetization transfer effects for the TSE readout scheme [26].

A limitation of this study is that the data were not collected with multiple post-label delay times to compute individual subject arterial transit delay time maps for use in the perfusion quantification; instead, a fixed arterial transit delay time was assumed. However, for all readout schemes, the same selective and non-selective label positions and widths were used, and the slice coverage was small at 2.5 cm; thus, arterial transit delay effects were minimal and equally affected each of the readout schemes in perfusion quantification.

These estimates form the basis for interpreting optimal readout schemes for guiding future study design in assessing ASL renal perfusion.

## 5. Conclusions

FAIR ASL measures were collected for bFFE, GE-EPI, SE-EPI, and TSE readout schemes. All schemes were found to be repeatable with coefficients of variation less than 29% for all techniques. When comparing all four techniques, we conclude that SE-EPI provides optimal temporal SNR, consistency across slices, and repeatability between sessions, and has the lowest specific absorption rate.

## Figures and Tables

**Table 1:** Imaging parameters for the balanced fast field echo (bFFE), gradient-echo echo-planar imaging (GE-EPI), spin-echo echo-planar imaging (SE-EPI), and turbo spin-echo (TSE) readout schemes. The post-label delay (PLD) is defined as the time to the central k-space for the first slice.

Readout Scheme	Post-Label Delay (ms)	Echo Time (ms)	Flip Angle (°)	Number of Slices (Slice Gap (mm))	Slice Spacing (ms)
bFFE	1300	1.5	45	5 (0)	280
GE-EPI	1800	8	90	5 (0)	40
SE-EPI	1800	18	90	5 (0)	60
TSE	1300	50	90	3 (5)	480

**Table 2:** Perfusion-weighted image signal-to-noise ratio (PWI-SNR), temporal SNR (tSNR), and variability of the perfusion-weighted signal (var_ΔM_) for each scheme.

Readout Scheme	PWI-SNR	tSNR	var_∆M_ (%)
bFFE	6.2 ± 3.6	2.4 ± 2.0	26 ± 11
GE-EPI	6.3 ± 1.5	1.5 ± 0.8	20 ± 5
SE-EPI	4.9 ± 1.5	2.6 ± 1.6	11 ± 3
TSE	8.5 ± 4.1	2.4 ±1.8	20 ± 4
